# Genome-wide CNV analysis replicates the association between *GSTM1* deletion and bladder cancer: a support for using continuous measurement from SNP-array data

**DOI:** 10.1186/1471-2164-13-326

**Published:** 2012-07-20

**Authors:** Gaëlle Marenne, Francisco X Real, Nathaniel Rothman, Benjamin Rodríguez-Santiago, Luis Pérez-Jurado, Manolis Kogevinas, Montse García-Closas, Debra T Silverman, Stephen J Chanock, Emmanuelle Génin, Núria Malats

**Affiliations:** 1 Spanish National Cancer Research Center (CNIO), Madrid, E-28029, Spain; 2 Inserm UMR-S946, Univ. Paris Diderot, Institut Universitaire d’Hématologie, Paris, F-75010, France; 3 Departament de Ciències Experimentals i de la Salut, Universitat Pompeu Fabra, Barcelona, E-08003, Spain; 4 Division of Cancer Epidemiology and Genetics, National Cancer Institute, Bethesda, MD, 20852-4907, USA; 5 Centro de Investigación Biomédica en Red de Enfermedades Raras (CIBERER), Barcelona, E-08003, Spain; 6 Programa de Medicina Molecular i Genètica, Hospital Universitari Vall d’Hebron, Barcelona, E-08003, Spain; 7 Department of Genome Sciences, University of Washington, Seattle, WA, 98195, USA; 8 Municipal Institute of Medical Research (IMIM-Hospital del Mar), Barcelona, E-08003, Spain; 9 Centre for Research in Environmental Epidemiology (CREAL), Barcelona, E-08003, Spain; 10 Centro de Investigación Biomédica en Red en Epidemiología y Salud Pública (CIBERESP), Barcelona, E-08003, Spain; 11 National School of Public Health, Athens, G-11521, Greece

**Keywords:** Bladder cancer risk, Glutathione *S*-transferase mu 1 (*GSTM1*), Copy number variation (CNV), SNP-array

## Abstract

**Background:**

Structural variations such as copy number variants (CNV) influence the expression of different phenotypic traits. Algorithms to identify CNVs through SNP-array platforms are available. The ability to evaluate well-characterized CNVs such as *GSTM1* (1p13.3) deletion provides an important opportunity to assess their performance.

**Results:**

773 cases and 759 controls from the SBC/EPICURO Study were genotyped in the *GSTM1* region using TaqMan, Multiplex Ligation-dependent Probe Amplification (MLPA), and Illumina Infinium 1 M SNP-array platforms. CNV callings provided by TaqMan and MLPA were highly concordant and replicated the association between *GSTM1* and bladder cancer. This was not the case when CNVs were called using Illumina 1 M data through available algorithms since no deletion was detected across the study samples. In contrast, when the Log R Ratio (LRR) was used as a continuous measure for the 5 probes contained in this locus, we were able to detect their association with bladder cancer using simple regression models or more sophisticated methods such as the ones implemented in the CNVtools package.

**Conclusions:**

This study highlights an important limitation in the CNV calling from SNP-array data in regions of common aberrations and suggests that there may be added advantage for using LRR as a continuous measure in association tests rather than relying on calling algorithms.

## Background

**Figure 1:**
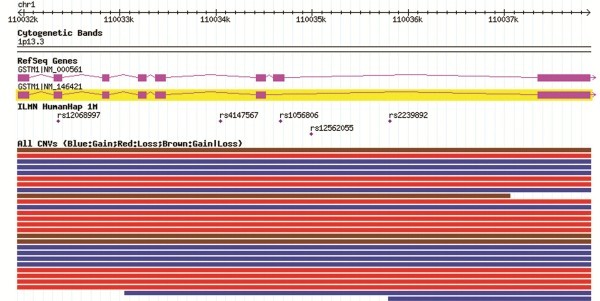
***GSTM1*locus and reported CNVs.** Source: Database of Genomic variation, accessed June 2011.

The glutathione *S*-transferase mu 1 (*GSTM1*) gene is located in the 1p13.3 band and codes for the cytosolic enzyme GST-μ that plays a role in carcinogen detoxification. Many structural variations have been described that overlap this gene in the Database of Genomic Variation [1] (Figure 1). A common copy number variant (CNV) has been well characterized and reported on the basis of the frequency of homozygous deletions of the entire coding region (known as the *GSTM1-*null genotype), which varies between 29% and 51% across ethnic groups [2-4].

Due to the established role of *GSTM1* in detoxification and the high frequency of homozygous deletions in the population, *GSTM1* has been extensively investigated in association with many chronic diseases, in particular, with asthma [5] and different types of cancers [4,6], including bladder cancer [7]. In a large case–control study, we reported that 63% of bladder cancer cases and 47% of controls harbored the *GSTM1*-null genotype, leading to an OR = 1.7 (95%CI 1.4 – 2.0) [8]. This is a well-established association as it was replicated in several independent studies and confirmed in a meta-analysis [8].

Reliable and accurate technologies such as qPCR or Multiplex Ligation-dependent Probe Amplification (MLPA) are available to genotype CNVs in targeted regions of the genome. High-throughput SNP-array platforms now offer the possibility to explore CNVs at a genome-wide scale. For instance, Illumina Infinium 1 M provides intensity data of both alleles at each SNP allowing the detection of CNV breakpoints and the estimation of the associated number of copies. Monomorphic probes were also included in genomic regions known to harbor CNVs but that were not well covered by SNPs. Overall, this platform contains 1,071,820 probes, among them 206,665 are located in reported CNV regions and 17,202 are monomorphic probes specially designed for CNV purpose.

Assessing CNV data across the genome-wide level using SNP-arrays is a daunting problem and requires stringent quality control (QC) measures to minimize the noise related to an analytical scheme that relies on sliding windows across SNPs [9-11]. The assessment also requires that CNVs is called based on the raw intensity data (Log-R ratio, LRR) from each probe. Several calling algorithms, such as PennCNV, have been developed [9,10,12,13] but these algorithms have a very low sensitivity when applied to large data sets [14-16]. In turn, the probability of false negatives remains a major challenge.

To limit false negative callings, a strategy that by-passes the calling step and directly performs the association test using LRR measures has been proposed [17-19]. Alternatively, methods have been developed allowing the simultaneous calling and association test estimation. These methods account better for the calling uncertainties but are yet to be validated in sufficiently large studies [20,21].

The objective of this study was to compare the assessment of a CNV at the *GSTM1* locus and its association with bladder cancer by applying LRR and PenCNV to data derived from the Illumina Infinium 1 M SNP-array platform with that derived from TaqMan (qPCR) and MLPA genotyping in subjects included in the Spanish Bladder Cancer/EPICURO (SBC/EPICURO) Study.

## Methods

### Samples

The SBC/EPICURO Study is a hospital-based case–control study conducted between 1998 and 2001 and described in detail elsewhere [8]. In summary, 18 hospitals in 5 Spanish regions (Barcelona, Vallès/Bages, Alicante, Asturias, Tenerife) participated in the Study with a total of 1,219 cases and 1,271 controls having been interviewed. Controls were matched to cases for gender, age and hospital. Detailed epidemiological information of known and potential risk factors for bladder cancer was collected. Genomic DNA was available for most of the individuals; and after exclusion based on DNA quality, tumor morphology and ethnicity [8], a total of 2,314 individuals (1,157 cases and 1,157 controls) were suitable for the genetic analysis.

Informed consent from all subjects and ethical approval from local and NCI, USA, institutional review boards were obtained.

#### *GSTM1* genotyping

Three genotyping methods were applied to assess the number of copies at the *GSTM1* locus. TaqMan assays were conducted at the Genotyping Core Facility – National Cancer Institute (CGF-NCI), USA, using the SNP500CancerID: GSTM1-02 probe [8]. MLPA assays were performed at the Pompeu Fabra University (UPF, Barcelona) by using optimized custom probes described elsewhere [22]. A genome-wide Illumina Infinium 1 M SNP-array scan was performed at the CGF-NCI, USA [7]. The latter platform provided information on 5 probes located in the the *GSTM1* locus (Figure 1).

While a different number of individuals were analyzed by each platform, a common set of 1,532 blood-derived samples (773 cases and 759 controls) was available for comparison. Details on the sample sets used for each genotyping platforms are provided in Additional file 1 Table S1 and Additional file 1 Figure S1.

### Retrieving CNV information from Illumina Infinium 1 M SNP-array

The Beadstudio software (Illumina Inc.) was used to process the data. Briefly, for each SNP probe, allele specific fluorescence intensities corresponding to the two alleles, named respectively A and B, were obtained and normalized to adjust for global differences in intensity and to scale the data as described in [23]. Genotype clusters have been calculated using our own data. The reliability of the SNP genotyping was evaluated through the analysis of duplicated samples; the observed concordance was >99.5%. The log R Ratio (LRR) was computed by taking the log2 value of the ratio of the sum of the normalized intensities, R_obs_, divided the value R_exp_ expected based on the genotype clusters. The LRR value depends on the number of CNV copies carried by the individual: typically for individuals belonging to the genotype clusters who carry only 2 copies (normal state), the LRR is around 0, while for individuals carrying less or more copies, the LRR is expected to be negative and positive, respectively. Beadstudio also computes the proportion of B alleles in the genotype, referred to as the B allele frequency or BAF, from the normalized intensities. For individuals carrying two copies, the BAF should be around 0, 0.5 or 1, depending on whether their genotypes are AA, AB, or BB, respectively.

For CNV calling, we used PennCNV [9] as, for our data set we obtained a better reliability (0.65) based on replicated samples for this algorithm in comparison to other two algorithms [16]. PennCNV implements a Hidden-Markov model (HMM) in which the hidden states are the number of copies (from 0 to 4 copies), and the observed states are the LRR and the BAF values at each probe. One of the HMM parameters is the vector of expected LRR values for each hidden state, the default values for 0, 1, 2, 3 and 4 copies are respectively −3.53, -0.66, 0, 0.40 and 0.68. According to PennCNV authors recommendations, we excluded all individual samples fulfilling at least one of the following criteria: a standard deviation of the LRR values over the 1 M probes > 0.28, a median BAF value out of the range [0.45 - 0.55], a BAF drift >0.002, a wave factor out of [−0.04 - 0.04]. The BAF drift summarizes the departure of the BAF from the expected values when 2 copies (0, 0.5 and 1). The wave factor aims to identify samples in which the LRR is not consistent across the genome; it summarizes the variability of the average LRR values in sliding windows.

### Statistical method for association testing with LRR

The association between *GSTM1* signal and bladder cancer risk was first tested using the LRR values. To this end, we used logistic regression models where, after adjustment for gender, age, region and tobacco consumption, the disease status was modeled as a function of the LRR at each of the five probes located in the gene (5 tests were performed). Second, we applied the association testing method implemented in the CNVtools package, which unifies genotyping and association testing into a single model by incorporating a dichotomous disease variable into the mixture model for the signal [20]. As recommended by the authors, the method was run using a summarized measure of the LRR across the 5 SNPs located in the *GSTM1* gene obtained after applying a principal component analysis and followed by a linear discriminant function. This analysis was also adjusted for gender, region, tobacco consumption and age. Since CNVtools only allows the adjustment for qualitative variables, we categorized the age in 4 classes according to its quartiles.

Analyses were performed using the statistical software R2.9. (http://www.r-project.org) and the Vennarable R package (Jonathan Swinton).

## Results

The concordance rate between TaqMan and MLPA for *GSTM1*-null identification was high but not complete: 96.2% over the entire sample (cases and controls) and 95.8% when considering only the controls. TaqMan and MLPA detected 402 (52.96%) and 401 (52.83%) controls carrying a homozygous deletion, respectively, and 289 controls (38.1%, both platforms) carrying a heterozygous deletion (Additional file 1 Table S2). These values were consistent with the reported rate of deletion in the European population. In contrast, when we conducted an analysis with PennCNV on the Illumina 1M SNP-array data, no deletion was detected among the 759 controls and 773 cases.

**Figure 2:**
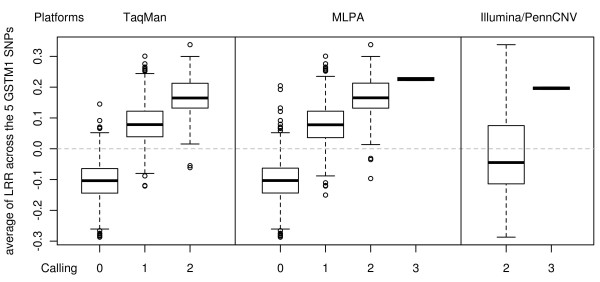
**LRR distribution according to CNV callings.** Average LRR from the 5 probes located at *GSTM1* according to the CNV calling obtained from TaqMan, MLPA and Illumina/PennCNV.

As shown in Figure 2, the average LRR values for the 5 Illumina probes located at the *GSTM1* locus was higher than expected based on the number of copies reported by TaqMan or MLPA. Indeed, for the individuals with two copies according to both TaqMan and MLPA, the LRR for the five probes was 0.17 on average, higher than 0, the value expected when 2 copies of the gene are present. Similarly, although negative LRR values are expected when a deletion is present, we observed positive values (0.08) in individuals with one copy and only slightly negative values (−0.10) in individuals with a homozygous deletion according to TaqMan and MLPA callings. These differences between the observed pattern of LRR at the *GSTM1* locus and the one expected by the PennCNV algorithm could explain that no detection was detected by PennCNV.

However, there was a strong correlation between LRR values and the estimated number of copies using TaqMan or MLPA (analysis of variance - p < 10^-15^), suggesting that the LRR values might be useful as an exploratory approach (Figure 2). Using LRR values from each of the five probes as explanatory variables in the logistic regression models, we detected the association with bladder cancer risk for three of them: (rs4147567: p = 8.00 × 10^-4^; rs2239892: p = 0.01 and rs12068997: p = 0.02) (Table 1). Estimated OR at the 5 SNPs (Table 1) was <1.0 indicating that an increase of the LRR was protective for bladder cancer, which was concordant with the evidence in the literature. Indeed, applying the callings provided by TaqMan and MLPA in this sample, we obtained a significant association (p = 3.40 × 10^-4^, OR = 0.74 [0.62-0.87] and p = 1.15 × 10^-4^, OR = 0.72 [0.61-0.85], respectively) between the trend on the number of copies at *GSTM1* and bladder cancer risk.

**Table 1:** **Association between*GSTM1*and bladder cancer**

	**Controls**		**Cases**			
**Data**	**N**	**average (std) of LRR**	**N**	**average (std) of LRR**	**OR [95%CI]**	**p-value**
rs12068997 (110,032,359)	756	−0.0091 (0.1066)	767	−0.0223 (0.1086)	0.30 [0.11-0.82]	0.0190
rs4147567 (110,034,047)	757	−0.0138 (0.2810)	767	−0.0642 (0.2661)	0.51 [0.35-0.76]	8.47 × 10^-4^
rs1056806 (110,034,670)	757	−0.0031 (0.1105)	767	−0.0096 (0.1061)	0.67 [0.25-1.80]	0.4279
rs12562055 (110,034,988)	756	−0.0001 (0.1266)	767	−0.0069 (0.1244)	0.83 [0.36-1.95]	0.6754
rs2239892 (110,035,809)	757	−0.0216 (0.2099)	767	−0.0474 (0.2043)	0.51 [0.30-0.85]	0.0103
**CNVtools**	756		767		0.66	1.74 × 10^-3^
**TaqMan**	757		767		0.74 [0.62-0.87]	3.40 × 10^-4^
**MLPA**	757		767		0.72 [0.61-0.85]	1.15 × 10^-4^

The association was performed by logistic regression models adjusted for age, gender, region and smoking status.

When using CNVtools to test for association, individuals were clustered into two categories based on their copy numbers. These categories fitted well with their actual status of having a homozygous deletion or not as detected by MLPA or TaqMan. We observed a high concordance rate, 93.7% and 93.0%, between CNVtools clustering and TaqMan and MLPA callings across the overall sample set. An association signal was detected (OR = 0.66, p = 1.74 × 10^-3^) using this method adjusting for age, gender, region and tobacco use, replicating the known association between *GSTM1* and bladder cancer.

## Discussion

Genome structural variants and, particularly CNV, are thought to play an important role in phenotypic variation and in the development of many complex diseases. In the last few years, several calling algorithms have been developed to identify CNVs at the whole genome scale using the same SNP-chips used to perform GWAS. However, studies that have evaluated the available tools have concluded that they lack sensitivity leading to a large number of false negative callings [14-16]. While PennCNV algorithm was found to be the one performing the best in previous comparisons, here we demonstrate the lack of sensitivity of PennCNV in a particular scenario. In the well-characterized region of *GSTM1*, we found that PennCNV did not detect any deletion in a large sample of cases with bladder cancer and controls where homozygous deletion was known to have a frequency of 50% using Taqman and MLPA technologies. Because PennCNV was designed to identify unknown CNV regions, we also applied the cnvHap algorithm that was designed to genotype known CNV regions [24]. As expected, cnvHap did not detect any deletion in the *GSTM1* region in our sample, either. It is noteworthy the fact that, using CNV calls derived from Illumina 1 M platform, *GSTM1* would have never been associated with bladder cancer. However, when individual probe LRR values are compared between cases and controls, the association can be detected and provide results similar to those obtained when using Taqman or MLPA. This observation clearly shows that PennCNV lacks sensitivity to detect CNV in the *GSTM1* region.

A possible explanation for the lack of sensitivity of PennCNV (and cnvHap) is the high frequency of the *GSTM1* deletion in the studied population. Indeed, CNV calling is done using the LRR that depends both on the observed (R_obs_) and the expected (R_exp_) R values. The R_exp_ is determined based on the clusters of genotypes. In the case of *GSTM1* where the homozygous deletion is very frequent, these clusters include a high number of subjects with a homozygous deletion (*GSTM1*-null genotype). Thus, R_obs_ and R_exp_ are expected to be similar in a *GSTM1*-null individual and, accordingly, the LRR value is around 0. The normalization process could also play a role as it aims at finding three clusters and this is not possible for *GSTM1* locus since the BAF of homozygous deleted sample is uniformly distributed between 0 and 1, thus normalization is affecting the intensity values, too.

The fact that the association between GSTM1 CNV and bladder cancer can be detected with LRR values without applying a calling CNV confirms the utility of this measure as a complementary screening strategy to test for association at the genome-wide level, as already suggested [17-19]. Indeed, the LRR is a continuous measure that approximates and correlates well with the actual discrete number of copies. Nonetheless, it is affected by the noise contained in the intensity measurement of both alleles obtained through the hybridization experiments. Thus, using LRR in the association test may decrease the power of some probes in detecting the association in comparison of using an accurate calling of the discrete number of copies. This loss of power would explain that two of the five probes located in the GSTM1 locus failed to show association with bladder cancer risk in our study, and that the three significant probes only showed a moderate significant p-values (between 8x10^-4^ and 0.019, Table 1). Nevertheless, even if the significance of these probes was moderate, we observed an excess of significant p-values in comparison to what we could expect under the null hypothesis of no association in that region. Thus, methods working at the genome-wide level and searching for regions with an excess of significant probes could have identified the GSTM1 region in our study. Alternatively, CNVtools, performing a joint calling and association testing, might also be considered, though it is more difficult to apply than that based on LRR and takes longer to run. The main caveat with CNVtools and equivalent methods is the definition of regions of interest.

The *GSTM1* deletion is located in a region of high sequence homology neighbored by a segmental duplication and this might explain that its breakpoints may slightly vary and, thus, the difficulties of calling. However, the locus is defined since the deletion in *GSTM1* was already known and approaches based in probes are able to identify it. Nevertheless, there might be other still unknown CNVs in the genome showing similar characteristics that might thus not be easy to call [25]. To increase sensitivity in CNV identification at the whole genome scale, we propose performing a genome-wide screen for association using LRR values at each probe and then applying CNVtools for a fine-tuning in the most promising regions.

## Conclusion

In conclusion, our study provides insights into the limitations of CNV-calling algorithms applied to SNP-array platforms in regions harboring common CNVs, especially those with full gene deletions. Though our results focused on a previously characterized CNV, they raise the possibility that there could be a substantial problem across unknown regions of the genome with common CNVs. On the other hand, we showed that by comparing LRR between cases and controls we were able to identify hot genomic areas associated with the trait of interest, supporting the use of this exploratory association assessment at the whole-genome level, which should be pursued with promising efficient calling algorithms.

## Competing interest

We declare we have no competing interest.

## Authors’ contributions

GM, SJC, NM and EG participated in the design of the study and drafted the manuscript. GM performed the statistical analysis. FXR, NR, BR, LP, MK, MG and DTS are involved in the SBCS study and help to draft the manuscript. All authors read and approved the final manuscript.

## Authors’ information

Co-last authors: Núria Malats, Emmanuelle Génin and Stephen J Chanock

## Supplementary Material

Additional file 1 **Table S1.** Details on the number of individuals genotyped by each of the three platforms. Number of callings available at *GSTM1* are also provided. **Table S2.***GSTM1* CNV assessment conducted by each of the genotyping platforms. We applied the PennCNV algorithm to call the CNVs from the Illumina 1M array genotyping data. **Figure S1.** Venn diagrams describing the common individuals genotyped by the three platforms a) for the cases and controls, b) for cases only and c) for controls only. Click here for file
