# Supplementary material for: Genome-wide CNV analysis replicates the association between GSTM1 deletion and bladder cancer: a support for using continuous measurement from SNP-array data
Source: BMC Genomics. 2012 Jul 20;13:326. doi: 10.1186/1471-2164-13-326 (PMC3425254; doi:10.1186/1471-2164-13-326)
Supplement: Additional file 1 — Table S1. Details on the number of individuals genotyped by each of the three platforms. Number of callings available at GSTM1 are also provided. Table S2.GSTM1 CNV assessment conducted by each of the genotyping platforms. We applied the PennCNV algorithm to call the CNVs from the Illumina 1M array genotyping data. Figure S1. Venn diagrams describing the common individuals genotyped by the three platforms a) for the cases and controls, b) for cases only and c) for controls only. [file 1471-2164-13-326-S1.doc]

**Supplementary table 1.** Details on the number of individuals genotyped by each of the three platforms. Number of callings available at *GSTM1* are also provided.

|  | **Cases** | **Controls** | **TOTAL** |
| --- | --- | --- | --- |
| **TaqMan** | | | |
| whole sample | 1157 | 1157 | 2314 |
| non-missing *GSTM1* call | 1145 | 1140 | 2285 |
| **MLPA on blood-derived samples** | | | |
| whole sample | 833 | 817 | 1650 |
| non-missing *GSTM1* call | 830 | 815 | 1645 |
| **Illumina / PennCNV** | | | |
| whole sample | 1113 | 1093 | 2206 |
| sample after quality control | 1056 | 1020 | 2076 |

**Supplementary table 2.** *GSTM1* CNV assessment conducted by each of the genotyping platforms. We applied the PennCNV algorithm to call the CNVs from the Illumina 1M array genotyping data.

|  | **Cases** | |  | **Controls** | |  |  |
| --- | --- | --- | --- | --- | --- | --- | --- |
|  | **N** | **%** |  | **N** | **%** |  | **TOTAL** |
| Total | 773 | 100% |  | 759 | 100% |  | 1532 |
| **TaqMan callings** |  |  |  |  |  |  |  |
| 0 copy | 488 | 63.1% |  | 402 | 53.0% |  | 890 |
| 1 copy | 232 | 30.0% |  | 289 | 38.1% |  | 521 |
| 2 copies | 53 | 6.9% |  | 68 | 9.0% |  | 121 |
| **MLPA callings** |  |  |  |  |  |  |  |
| 0 copy | 491 | 63.5% |  | 401 | 52.8% |  | 892 |
| 1 copy | 229 | 29.6% |  | 289 | 38.1% |  | 518 |
| 2 copies | 53 | 6.9% |  | 67 | 8.8% |  | 120 |
| 3 copies | 0 | 0% |  | 2 | 0.3% |  | 2 |
| **Illumina / PennCNV callings** |  |  |  |  |  |  |  |
| 2 copies | 772 | 99.9% |  | 758 | 99.9% |  | 1530 |
| 3 copies | 1 | 0.1% |  | 1 | 0.1% |  | 2 |

**Supplementary figure 1.** Venn diagrams describing the common individuals genotyped by the three platforms a) for the cases and controls, b) for cases only and c) for controls only.
